# Unconscious Processing of Facial Expressions in Individuals with Internet Gaming Disorder

**DOI:** 10.3389/fpsyg.2017.01059

**Published:** 2017-06-23

**Authors:** Xiaozhe Peng, Fang Cui, Ting Wang, Can Jiao

**Affiliations:** ^1^College of Psychology and Sociology, Shenzhen UniversityShenzhen, China; ^2^Shenzhen Key Laboratory of Affective and Social Cognitive Science, Shenzhen UniversityShenzhen, China; ^3^Faculty of Humanities and Social Science, City University of MacauMacau, China

**Keywords:** Internet Gaming Disorder, backward masking, unconscious facial processing, ERPs, N170

## Abstract

Internet Gaming Disorder (IGD) is characterized by impairments in social communication and the avoidance of social contact. Facial expression processing is the basis of social communication. However, few studies have investigated how individuals with IGD process facial expressions, and whether they have deficits in emotional facial processing remains unclear. The aim of the present study was to explore these two issues by investigating the time course of emotional facial processing in individuals with IGD. A backward masking task was used to investigate the differences between individuals with IGD and normal controls (NC) in the processing of subliminally presented facial expressions (sad, happy, and neutral) with event-related potentials (ERPs). The behavioral results showed that individuals with IGD are slower than NC in response to both sad and neutral expressions in the sad–neutral context. The ERP results showed that individuals with IGD exhibit decreased amplitudes in ERP component N170 (an index of early face processing) in response to neutral expressions compared to happy expressions in the happy–neutral expressions context, which might be due to their expectancies for positive emotional content. The NC, on the other hand, exhibited comparable N170 amplitudes in response to both happy and neutral expressions in the happy–neutral expressions context, as well as sad and neutral expressions in the sad–neutral expressions context. Both individuals with IGD and NC showed comparable ERP amplitudes during the processing of sad expressions and neutral expressions. The present study revealed that individuals with IGD have different unconscious neutral facial processing patterns compared with normal individuals and suggested that individuals with IGD may expect more positive emotion in the happy–neutral expressions context.

**Highlights:**

• The present study investigated whether the unconscious processing of facial expressions is influenced by excessive online gaming. A validated backward masking paradigm was used to investigate whether individuals with Internet Gaming Disorder (IGD) and normal controls (NC) exhibit different patterns in facial expression processing.
• The results demonstrated that individuals with IGD respond differently to facial expressions compared with NC on a preattentive level. Behaviorally, individuals with IGD are slower than NC in response to both sad and neutral expressions in the sad–neutral context. The ERP results further showed (1) decreased amplitudes in the N170 component (an index of early face processing) in individuals with IGD when they process neutral expressions compared with happy expressions in the happy–neutral expressions context, whereas the NC exhibited comparable N170 amplitudes in response to these two expressions; (2) both the IGD and NC group demonstrated similar N170 amplitudes in response to sad and neutral faces in the sad–neutral expressions context.
• The decreased amplitudes of N170 to neutral faces than happy faces in individuals with IGD might due to their less expectancies for neutral content in the happy–neutral expressions context, while individuals with IGD may have no different expectancies for neutral and sad faces in the sad–neutral expressions context.

## Introduction

Excessive computer game playing can be both addictive and pathological (D’Hondt et al., 2015; Lemmens et al., 2015). As a behavioral addiction, Internet Gaming Disorder (IGD) is characterized by compulsive gaming behaviors with harmful personal or social consequences, such as impairments in individuals’ academic, occupational, or social functioning (Brady, 1996; Young, 1998; DSM-V, American Psychiatric Association, 2013; Tam and Walter, 2013; Spada, 2014; Van Rooij and Prause, 2014; D’Hondt et al., 2015; Kuss and Lopez-Fernandez, 2016). Research has found that Internet addiction (including online gaming activities and other forms of Internet use) shares essential features with other addictions, including decreased executive control abilities and excessive emotional processing of addiction-related stimuli (Ng and Wiemer-Hastings, 2005; He et al., 2011; D’Hondt et al., 2015). Accordingly, previous studies of IGD focused predominantly on impairments in inhibitory control or executive control among individuals with IGD (Dong et al., 2010, 2011; Wang et al., 2013; D’Hondt et al., 2015; Zhang et al., 2016). The deficits of individuals with IGD in social interactions and social skills such as emotional and interpersonal communication have also received considerable attention (Young, 1998; Engelberg and Sjöberg, 2004; D’Hondt et al., 2015), but so far, there have been limited experimental studies on the processing of real-world socioemotional stimuli among individuals with IGD. Thus, the underlying mechanisms behind these deficits remain unclear.

Social communication has been suggested to depend largely on the capacity for expression recognition (Blair, 2005; He et al., 2011). Facial expressions are important socioemotional stimuli, as they can convey information about the identities, emotions, and intentions of other people, and thus represent a primary element of non-verbal communication in everyday life (Batty and Taylor, 2003; Itier and Taylor, 2004). Previous studies indirectly related to facial processing in IGD found that action video game players or violent media users had a reduced attention to happy faces in emotion recognition tasks (Kirsh et al., 2006; Kirsh and Mounts, 2007; Bailey and West, 2013). For example, Kirsh et al. (2006) found that compared with participants low in violent media consumption, participants high in violent media consumption were slower to identify happy expressions and faster to identify anger expressions. However, IGD-afflicted individuals’ processing of facial expressions remains unclear. Furthermore, studies on normal participants have revealed that emotional cues can be extracted from facial expressions in the preattentive or unconscious stage of face processing (Öhman, 1999; Dimberg et al., 2000; Vuilleumier and Schwartz, 2001; Vuilleumier, 2002). However, although deficits in conscious neutral-face processing were found in excessive Internet users (He et al., 2011), whether individuals with IGD had unique unconscious emotional facial processing patterns remained unclear. We therefore aimed to explore this issue in the present study.

To further investigate unconscious facial processing in individuals with IGD, the present study employed a visual backward masking paradigm. Visual backward masking is an “empirically rich and theoretically interesting phenomenon” that indicates the attenuation of the visibility of a target stimulus by a mask stimulus presented after the target (Breitmeyer, 1984; Breitmeyer and Ogmen, 2000, p. 1572). In this paradigm, a target stimulus is presented briefly (usually for 1–100 ms) and followed by a mask stimulus, which is a meaningless or scrambled picture that overlaps with the target stimulus spatially or structurally (Esteves and Öhman, 1993). The mask stimulus impairs the explicit awareness or perception of the target stimulus (Morris et al., 1999; Breitmeyer and Ogmen, 2000). This paradigm has been widely used to investigate recognition thresholds as well as to examine emotional and visual information processing, which are partially independent of awareness, in a variety of specific subject populations, such as people with affective disorders (Esteves and Öhman, 1993; Breitmeyer and Ogmen, 2000; Axelrod et al., 2015; Zhang et al., 2016). For example, Zhang et al. (2016) found deficits of unconscious facial processing in patients with major depression using the visual backward masking paradigm with event-related potentials (ERPs).

To gain a better understanding of unconscious facial processing, we used ERPs, which have high temporal resolution, in the present study. To our knowledge, there was only one published ERP study focusing on the facial processing of excessive Internet users (He et al., 2011). He et al. (2011) found deficits in early face processing among excessive Internet users by asking participants to passively view upright and inverted faces and non-face stimuli presented above the conscious threshold. Specifically, excessive Internet users were found to be impaired in social stimulus processing but intact in holistic configural face processing, which were represented as a smaller N170 face effect (i.e., the difference in the amplitudes of the N170 for neutral-face vs. non-face stimuli) and similar N170 inversion effect (i.e., the difference in the amplitudes of the N170 component of ERP in response to upright vs. inverted neutral faces) in excessive Internet users compared with normal controls (NC; He et al., 2011). N170 is widely acknowledged to be a face-sensitive ERP component, typically occurring 140 to 200 ms after stimulus onset and responding maximally to face stimuli, reflecting automatic processing in the early stage of face perception (Rossion et al., 2000; Itier and Taylor, 2004). The N170 component has been found to be not only associated with the structural encoding of faces (e.g., Dimberg et al., 2000; Sato et al., 2001; Eimer et al., 2003; Holmes et al., 2003; Schupp et al., 2004), but also modulated by emotional facial expressions (e.g., Blau et al., 2007; Pegna et al., 2008; for review, see Rellecke et al., 2013). Third, N170 was found to be associated with unconscious face processing in normal subjects (e.g., Pegna et al., 2008; Carlson and Reinke, 2010). For example, using the backward masking paradigm, Carlson and Reinke (2010) found that a masked fearful face enhanced the contralateral N170. Thus, in the present study, the N170 amplitude was taken as the index which indicated unconscious emotional facial perception in the early stage of face processing. Furthermore, expectancies for emotional content were suggested to influence the recognition of facial expressions (Leppänen et al., 2003; Hugenberg, 2005). For example, facilitation of the processing was observed when the stimuli were congruent with participants’ expectancies, and the opposite effect was observed when the stimuli were incongruent with participants’ expectancies (Leppänen et al., 2003; Hugenberg, 2005). Besides, according to a cognitive-behavioral model of problematic Internet use, pathological involvement in gaming results from problematic cognitions coupled with behaviors maintaining maladaptive responses (Davis, 2001). For example, individuals who have negative views of themselves may use gaming to achieve positive social interactions, social acceptance, or positive social feedback (King and Delfabbro, 2014). Besides, previous study found that individuals with Internet addiction had higher scores on the Behavior Inhibition System and Behavior Approach System Scale (BIS/BAS scale) fun-seeking subscales, suggesting that these individuals had higher sensitivity to the stimuli with reward, and were more likely to engage in approach behavior for the rewarding stimuli (Yen et al., 2009). Based on these previous findings which indicated the influence of expectancy on facial expression recognition (Leppänen et al., 2003; Hugenberg, 2005), together with the association between problematic gaming behavior with individuals with IGD and their aforementioned social needs (King and Delfabbro, 2014), and IGD’s higher sensitivity to rewarding stimuli (Yen et al., 2009), we speculate that to individuals with IGD, neutral faces are comparatively less rewarded than happy faces; accordingly, individuals with IGD may have less expectancy for neutral stimuli than for positive stimuli, and this incongruence would subsequently led to the lower activation for neutral expressions than happy expressions. Thus, we expected to observe that IGD show reduced N170 amplitudes in response to neutral expressions in the happy–neutral context, while NC group show comparable N170 to happy and neutral expressions in the happy–neutral context, which may represent different patterns in emotional facial processing between individuals with IGD and NC. Whereas this effect would not present in the sad–neutral context since individuals in both groups have no expectancy for sad or neutral expressions.

## Materials and Methods

### Participants

Sixteen participants with IGD and 16 NC were recruited from local universities in Shenzhen, China. Descriptions of participants’ demographics are presented in **Table 1**. There were no significant differences between the two groups in terms of age, handedness, or education. The proposed diagnostic cutpoint of the DSM-5 was suggested to be conservative (e.g., Lemmens et al., 2015); thus, Young’s Internet Addiction Test (IAT) was used to screen people for IGD in the present study. IAT is a reliable instrument and widely used in studies investigating Internet addiction disorders, including IGD (e.g., Khazaal et al., 2008). Young (1998) suggested that a score between 40 and 69 signifies problems due to Internet use. However, IAT relies on subjective ratings and is therefore susceptible to participants’ concealment or underestimation. Additionally, previous studies used “experience in playing video games of 10 or more hours a week” (Weinreich et al., 2015, p. 61) or “at least 4 years and for at least 2 h daily” (Szycik et al., 2017, p. 2) as the inclusion criterion for the expert/excessive users of violent video games. Thus, the present study also included the length of time that the participants spent on online gaming as a criterion. Individuals were asked to provide the number of hours per day and per week they spent online gaming. Individuals with score ≥40 on the IAT and who spent ≥4 h per day and ≥30 h per week on Internet gaming were included in our IGD cohort. Moreover, to control for comorbidities such as depression and anxiety (Sanders et al., 2000; Yen et al., 2007; Wei et al., 2012; Lai et al., 2015), we excluded individuals with IGD who scored more than 40 points on either the Zung Self-Rating Depression Scale (SDS) (Zung, 1965) or the Zung Self-Rating Anxiety Scale (SAS) (Zung, 1971). None of the participants had a history of head injury, neurological disorders, substance abuse or dependence over the past 6 months. All research procedures were approved by the Medical Ethical Committee of Shenzhen University Medical School according to the Declaration of Helsinki. All the participants provided written informed consent indicating that they fully understood the study.

**Table 1:** Participants’ demographics for the normal controls and individuals with IGD.

Characteristics	Control (*n* = 16)	IGD (*n* = 16)	*t*	*p*
Mean age (years)	20.25 ± 0.4	20.75 ± 0.36	-1.14	0.27
Gender (female/male)	4/12	3/13		
IAT	33.63 ± 1.3	58.13 ± 2.81	-7.33	0.00
SAS	27 ± 1.14	30.63 ± 1.31	-1.22	0.09
SDS	32.31 ± 0.98	33.94 ± 0.94	-1.18	0.26


*Descriptive data are presented as mean ± standard error. IAT, Internet Addiction Test (Young, 1998); SAS, Self-Rating Anxiety Scale (Zung, 1971); SDS, Self-Rating Depression Scale (Zung, 1965). Df is 15 in the statistics.*

### Stimuli

We used the backward masking task program (see Procedure) and stimuli employed in Zhang et al.’s (2016) study. The target face stimuli, including 20 happy expressions, 20 sad expressions, and 40 neutral expressions, were selected from the native Chinese Facial Affective Picture System (CFAPS), which includes pictures assessed by Chinese participants in a previous study (Gong et al., 2011). The above-mentioned study found significant differences in nine-point-scale ratings for both emotional valence and arousal among the three categories of expressions. The study reported the following for valence ratings: “(2,77) = 143, *p* < 0.001, = 0.787, happy = 5.92 ± 0.13; sad = 2.78 ± 0.13; neutral = 4.22 ± 0.09; pairwise comparisons: *ps* < 0.001; for arousal ratings, (2,77) = 30.2, *p* < 0.001, = 0.439, happy = 5.13 ± 0.22; sad = 5.83 ± 0.22; neutral = 3.82 ± 0.16; for pairwise comparisons, emotional vs. neutral: *p* < 0.001, happy vs. sad: *p* < 0.087” (Zhang et al., 2016, p. 15). The stimulus display and behavioral data acquisition were conducted using E-Prime software (version 2.0, Psychology Software Tools, Inc., Boston, MA, United States).

### Procedure

The procedure consisted of a happy block and a sad block. At the beginning of each trial, a central fixation cross was presented for 500 ms, followed by a 400-600 ms blank screen. Then, a target (happy/sad or neutral) face was presented for 17 ms, followed immediately by a scrambled face as a mask, which lasted for 150 ms (Zhang et al., 2016). Previous studies set the duration of the mask stimulus at 100 to 300 ms or other durations above the awareness threshold (e.g., Rolls and Tovee, 1994; Whalen et al., 1998; Fisch et al., 2009; for review, see Pessoa, 2005). Here, we used 150 ms according to the parameter in Zhang et al.’s (2016) study. The participants were required to discriminate the target faces by pressing two buttons on the computer keyboard with their left or right index fingers as soon as possible (Zhang et al., 2016). Each block included 160 trials with 80 emotional expressions and 80 neutral expressions that were randomized and presented as target stimuli—that is, 20 happy and 20 neutral faces were presented a total of four times in the happy block; 20 sad and 20 neutral faces were presented a total of four times in the sad block. The assignment of keys to each valence of expressions, and the sequence of blocks was counterbalanced across the participants (Zhang et al., 2016).

### ERP Recording

Brain electrical activity was recorded through a 64-electrode scalp cap using the 10–20 system (Brain Products, Munich, Germany). The TP10 channel was used as the reference during the recordings (Kaufmann et al., 2009; Ferrante et al., 2015; Cui et al., 2017). Two electrodes were used to measure the electrooculogram (EOG). EEG and EOG activity were amplified at 0.01–100 Hz passband and sampled at 500 Hz. The EEG data were recorded with all electrode impedances maintained below 5 kΩ. The EEG data from each electrode were re-referenced to the average of the left and right mastoids prior to further analysis.

The EEG data were pre-processed and analyzed using BrainVision Analyzer 2.1 (Brain Products, Munich, Germany). Pre-processing included bad channel detection and removal, epoching, and eyeblink removal. Then, the signal was passed through a 0.01–30 Hz band-pass filter. The epochs consisted of the 200 ms before and 1000 ms after the onset of the target stimuli. EOG artifacts were corrected using independent component analysis (ICA) (Jung et al., 2001). Epochs with amplitude values exceeding ±80 μV at any electrode were excluded before the application of the EEG averaging procedure. The ERPs were independently computed for each participant and each experimental condition.

The ERP was time-locked to the presentation of the target face. Based on previous research on face processing (Luo et al., 2010; Frühholz et al., 2011; Zhang et al., 2016) and the topographical distribution of the grand-averaged ERP activity in the current study, the average amplitudes at the P8 and PO8 electrode sites were selected for the statistical analysis of the N170 component (time window: 150–230 ms). For each component, mean amplitudes were obtained within the corresponding time window and averaged from the electrodes.

### Data Analysis

Further statistical analyses were conducted using IBM SPSS Statistics 22 (IBM Corp., Armonk, NY, United States). Because the happy and sad blocks were different emotional contexts, separate analyses of variance (ANOVAs) of the interaction of emotional valence (happy vs. neutral, sad vs. neutral, or happy vs. sad) × group (IGD vs. control) were conducted for the behavioral data and each ERP component. Both the behavioral data and the ERP amplitudes were analyzed with repeated measures ANOVAs using Greenhouse–Geisser adjusted degrees of freedom. The between-subject factor was the study group (IGD vs. control), and the within-subject factor was emotional valence of expression (happy vs. neutral, sad vs. neutral, or happy vs. sad). The *post hoc* analysis used Bonferroni corrections for multiple comparisons.

## Results

The numbers of trials included in the experimental conditions are listed in **Table 2**. For the following results, the descriptive data are presented as mean ± standard error unless noted otherwise.

**Table 2:** Number of trials included in each condition.

Condition	Control (*n* = 16)	IGD (*n* = 16)	*t*	*p*
Happy expressions	62.44 ± 3.29	55.44 ± 4.20	1.40	0.18
Neutral expressions in happy block	52.88 ± 4.78	54.5 ± 4.12	-0.36	0.73
Sad expressions	59.5 ± 2.60	51.2 ± 4.79	1.72	0.10
Neutral expressions in sad block	52.38 ± 3.74	46.75 ± 3.95	1.42	0.18


*Df is 15 in the statistics.*

### Behavioral Data

Regarding reaction time, in the sad block, the main effect of valence was significant, *F*(1,30) = 4.86, *p* < 0.05, = 0.14; reaction time was shorter for sad expressions (618.87 ± 31.48 ms) than for neutral expressions (663.39 ± 34.77 ms); the main effect of group was significant, *F*(1,30) = 5.09, *p* < 0.05, = 0.15; and reaction time was shorter for the NC group (569.84 ± 44.68 ms) than for the IGD group (712.42 ± 44.68 ms). The interaction was not significant, *p* > 0.5. In the happy block, the main effect of valence was significant, *F*(1,30) = 6.63, *p* < 0.05, = 0.18; reaction time was shorter for happy expressions (583.97 ± 39.33 ms) than for neutral expressions (648.08 ± 36.6 ms); no other main and interaction effects reached significance, all *p*s > 0.1; reaction time for the NC group (577.25 ± 50.76 ms) was comparable with that for the IGD group (654.81 ± 50.76 ms). When the happy and sad trials were directly compared, the main effect and the interaction were not significant, all *p*s > 0.05.

In terms of accuracy, in the sad–neutral block, in the happy–neutral block, and when the happy and sad trials were directly compared, no main effect and interaction effect reached significance.

### ERP Data

#### N170

**FIGURE 1:**
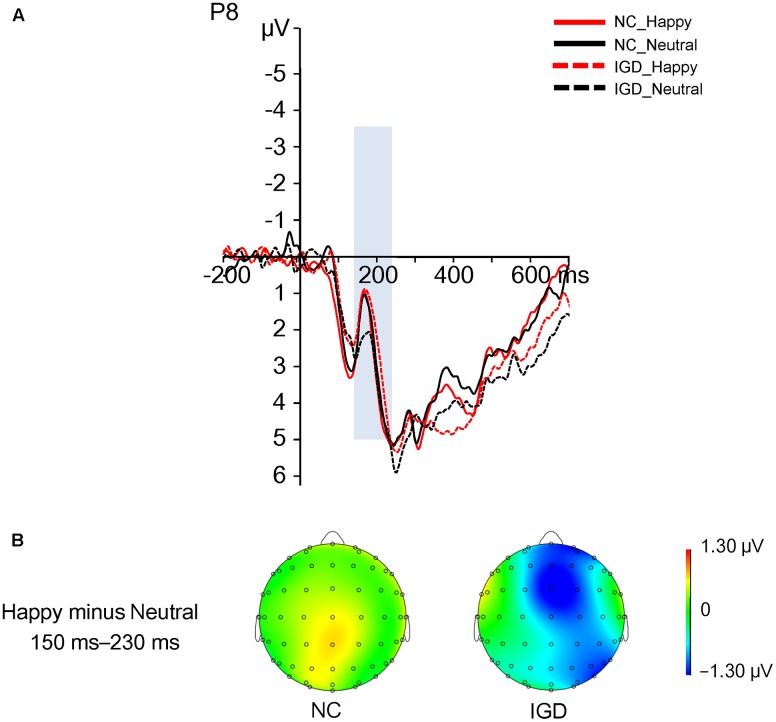
**(A)** Grand ERP waveforms of the N170 component displayed between 150 and 230 ms for the four conditions at the representative P8 site. **(B)** Topographic distributions of the difference waves between neutral and happy expressions (happy condition minus neutral condition) in the IGD and NC groups, with the 150–230 ms time interval.

A 2 (group) × 2 (happy vs. neutral) ANOVA revealed that the main effect of valence was not significant, (1,30) = 3.47, *p* = 0.07, = 0.10, and the main effect of group was not significant, (1,30) = 0.01, *p* = 0.92, < 0.001. However, the interaction of valence by group was significant, (1,30) = 4.25, *p* = 0.048, = 0.124 (**Figure 1**). The *post hoc* analysis revealed that for the IGD group, happy expressions elicited a comparatively more negative-directed N170 component (3.02 ± 1.12 μV) than neutral faces (4.18 ± 1.09 μV), (1,30) = 7.70, *p* = 0.009, = 0.20, Bonferroni corrected. However, for the control group, happy and neutral expressions elicited similar N170 components (happy: 3.79 ± 1.12 μV, neutral: 3.73 ± 1.09 μV), (1,30) = 0.02, *p* = 0.89, = 0.001, Bonferroni corrected.

**FIGURE 2:**
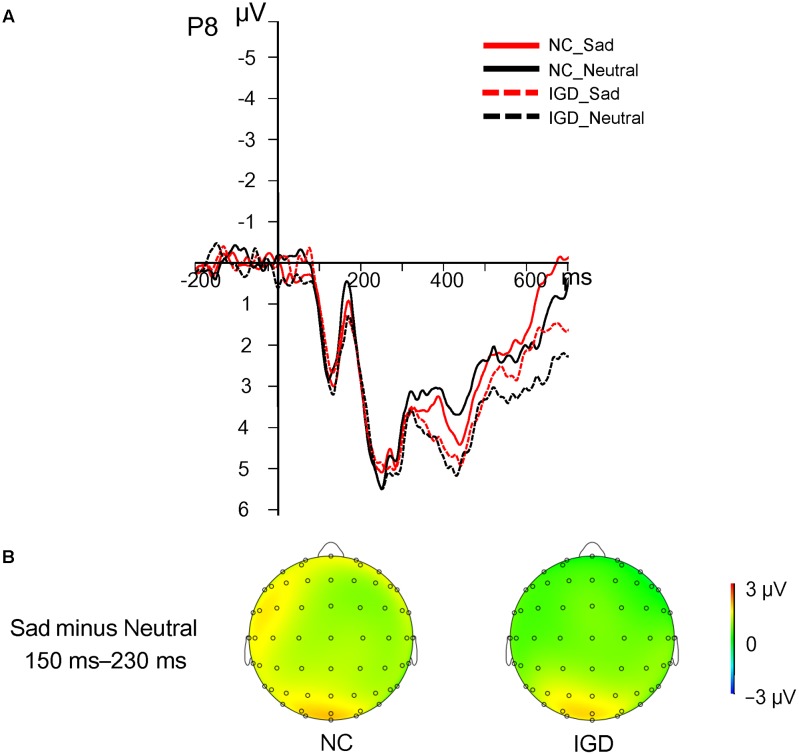
**(A)** Grand ERP waveforms of the N170 component displayed between 150 and 230 ms for the four conditions at the representative P8 site. **(B)** Topographic distributions of the difference waves between neutral and happy expressions (sad condition minus neutral condition) in the IGD and control groups, with the 150–230 ms time interval.

However, the amplitudes in the sad–neutral context did not show significant main or interaction effects in the sad–neutral condition (**Figure 2**). A 2 (group) × 2 (sad vs. neutral) ANOVA revealed that the main effects of valence [*F*(1,30] = 0.39, *p* = 0.54, = 0.01], group [*F*(1,30) = 0.02, *p* = 0.88, = 0.001], and the interaction [*F*(1,30) = 0.02, *p* = 0.88, = 0.001] were not significant and that the N170 components elicited by happy and neutral expressions in the IGD group (sad: 3.79 ± 1.21 μV, neutral: 3.65 ± 1.15 μV) were similar to those elicited in the control group (sad: 3.57 ± 1.21 μV, neutral: 3.35 ± 1.15 μV).

When directly comparing N170 amplitudes in response to sad and happy expressions, a 2 (IGD vs. NC group) × 2 (sad vs. happy) ANOVA demonstrated that the main effects of valence, group, and the interaction were not significant, all *p*s > 0.05.

## Discussion

As a perceptual basis for social interaction, emotional expression processing is an important component of interpersonal communication. Although a wealth of studies have investigated executive functions in individuals with IGD, studies on the emotional expression processing of individuals with IGD have been limited; in particular, to our knowledge, there have been no published studies investigating unconscious processing of emotional expressions in IGD. The behavioral data of the present study revealed that both the IGD and NC groups responded faster to unconscious emotional expressions (happy and sad expressions) than to neutral expressions, suggesting that individuals with IGD have normal ability to extract emotional signals from facial expressions in the preattentive stage. This result was consistent with a previous finding which demonstrated a shorter reaction time to emotional expressions than to neutral expression in normal participants (Calder et al., 1997; Eimer et al., 2003) and extended this finding to individuals with IGD. Besides, compared with IGD, NC group showed shorter reaction time to both sad and neutral expressions in the sad block. However, there was no similar effect on happy and neutral expressions in the happy block. Prototypical happy faces were suggested to be more easily recognized and more distinguishable from neutral than sad faces (Calder et al., 1997; Surguladze et al., 2003). Based on this suggestion, in the happy block, happy expressions might be more distinguishable than neutral expressions for both NC and IGD group, thus facilitate the recognition task for the two expressions in both NC and IGD group. While there was no facilitation of recognition in the sad block since the sad expressions are not much distinguishable from neutral expressions as happy expressions. These results suggest that regarding the reaction time, the sad block condition/sad–neutral context might be more sensitive in distinguishing IGD and NC in unconscious facial recognition.

More importantly, the present study explored the time course of unconscious emotional facial processing in individuals with IGD. The ERP results showed reduced N170 amplitude in individuals with IGD when they processed unconscious neutral faces compared with happy faces, while NC showed similar N170 amplitudes when they processed neutral and happy faces in the happy–neutral context. Both individuals with IGD and NC showed similar N170 amplitudes to sad faces and neutral faces in the sad–neutral context. The decreased N170 amplitude for neutral expressions compared to happy expressions in the IGD group support our hypothesis, which suggested that participants’ different expectancies in processing positive and negative stimuli would influence their facial recognition, and lead to different facial processing in IGD and NC. Participants’ expectancies were previously suggested to influence implicit evaluation by affecting the valence of the prime stimuli in the affective priming task (Leppänen et al., 2003; Hugenberg, 2005). In the present study, neutral expressions were less rewarded than happy expressions in individuals with IGD, and IGD might have less expectancy for neutral expressions than for happy expressions, resulting in decreased N170 amplitudes for neutral expressions than happy expressions. However, in the sad–neutral condition, individuals may not have more expectancy for sad faces or less expectancy for neutral faces, leading to similar responses to sad and neutral faces. It should be noted that we cannot conclude that individuals with IGD have deficits in emotional facial recognition, since they showed similar N170 amplitudes to those of NC in response to happy and sad expressions. On the other hand, this result implies that individuals with IGD may have normal ability to extract emotional information from emotional expressions. Furthermore, the present ERP data showed differences between IGD and NC group in happy block condition, while the behavioral data showed differences of two groups in sad block condition. We suggest that N170 represent the distinct unconscious face processing of IGD in early stage, whereas the reaction time might reflect the facial expressions recognition in the late stage. However, considering that behavioral data often does not align to ERP data for easy explanations, more studies are needed for this issue.

In summary, the present results extended the previous findings on the face processing of excessive Internet users and demonstrated distinct mechanisms for facial expression processing in different facial contexts among individuals with IGD. Specifically, compared with NC, individuals with IGD have lower N170 amplitudes in response to neutral faces than in response to happy faces in the happy–neutral expression context, which may arise from their lower expectancy for neutral expressions. This effect was not observed in the sad–neutral expression context for either IGD or NC individuals.

### Limitations and Future Studies

There are two limitations in the present study. First, more males than females were recruited due to the relative scarcity of females with excessive use of Internet game playing. Second, although previous studies found that substantial amounts of time in the virtual world (e.g., playing video games) were associated with individuals’ decreased interpersonal relationships in the real world and suggested that the lower frequency of social-emotional communication may alter how individuals with IGD process facial expressions in the real world (Lo et al., 2005; Weinreich et al., 2015), we cannot draw any conclusions about the causality of IGD subjects’ distinct facial expression processing pattern or the their impairments in social communication. More studies are needed to investigate the emotional facial processing mechanisms of individuals with IGD.

## Author Contributions

XP, FC, and CJ developed the concepts for the study. TW collected the data. XP and TW analyzed the data. XP, CJ, and FC wrote the manuscript. All authors contributed to the manuscript and approved the final version of the manuscript for submission.

## Conflict of Interest Statement

The authors declare that the research was conducted in the absence of any commercial or financial relationships that could be construed as a potential conflict of interest.
